# Moving past the face mask? Nasopharyngeal tube and aeration during preterm resuscitation

**DOI:** 10.1038/s41390-024-03127-1

**Published:** 2024-03-05

**Authors:** David M. Rub, Lucy Loft, David G. Tingay, Kate Hodgson

**Affiliations:** 1 Division of Neonatology, Children’s Hospital of Philadelphia, Philadelphia, PA USA; 2 Department of Pediatrics, Perelman School of Medicine, University of Pennsylvania, Philadelphia, PA USA; 3 Neonatal Research, Murdoch Children’s Research Institute, Melbourne, VIC Australia; 4 Neonatal Research, Royal Women’s Hospital, Melbourne, VIC Australia; 5 Department of Paediatrics, University of Melbourne, Melbourne, VIC Australia; 6 Department of Obstetrics, Gynaecology and Newborn Health, University of Melbourne, Melbourne, VIC Australia

At birth, very preterm infants must undergo physiological changes to clear the airway of foetal lung fluid, establish a functional residual capacity (FRC) and initiate pulmonary gas exchange. In very preterm infants, the combined effects of surfactant deficiency, immature lungs, chest wall weakness and reduced respiratory drive result in a high risk of requiring respiratory support at birth. Historically, endotracheal intubation and mechanical ventilation were standard practice for delivery room stabilisation of very preterm infants failing initial bag and mask support. Although life-saving, endotracheal intubation is a difficult technical skill for clinicians to perform,^1^ and mechanical ventilation is associated with poorer outcomes in preterm infants, including long-term respiratory and neurodevelopmental morbidities.^2^ Clinicians therefore attempt to avoid mechanical ventilation if possible, and support delivery room transition without endotracheal intubation.

The face mask is the most commonly used interface to provide respiratory support to neonates in the delivery room. However mask leak, airway obstruction and irregular tidal volume delivery may impact its effectiveness.^3^ Face mask application may also induce the trigeminocardiac reflex resulting in apnoea and bradycardia.^4^ Alternative neonatal airways have been investigated over recent years. While the laryngeal mask airway (LMA) is now recommended as a safe alternative to face mask ventilation for infants >34 weeks’ gestation, equipment size limits its utility in very preterm infants.^5^ The use of a nasopharyngeal tube (NPT) has also been explored as an alternative to the face mask as a primary respiratory support interface after birth. Important short-term clinical outcomes appear to be similar,^6^ however the NPT may result in greater leak and airway obstruction, with subsequent lower tidal volume delivery.^7^ Studies to date have not examined the NPT as a rescue device in very preterm infants not responsive to initial face mask ventilation.

In this issue of *Pediatric Research*, Belting et al. report a secondary analysis of a single centre, randomised controlled trial of preterm infants born 26–31 + 6 weeks’ gestation, evaluating the use of a rescue NPT in infants non-responsive to initial non-invasive ventilation after birth.^8^ The authors assess the change in lung volumes and cardiorespiratory parameters using respiratory function monitoring and electrical impedance technology (EIT). Fifteen patients, and 1154 inflations were included in this analysis. Average end-expiratory lung impedance (EELI) increased after the insertion of the NPT compared with EELI when mask support was commenced as the first line interface after birth, with the biggest volume changes being related to the transition from mask support to inserting and then commencing NPT ventilation. A similar increase was seen during initial face mask ventilation. Physiological parameters including SpO_2_/FiO_2_ ratio and heart rate improved significantly after the insertion of the NPT; eight of nine bradycardic infants normalised their heart rate.

Of note, the choice of delivered pressure, and the decisions to provide non-invasive positive pressure ventilation or to change the airway adjunct were at clinician discretion. Clinicians using the NPT were also afforded the use of a respiratory function monitor, whereas those using a face mask were not. Importantly, similar to many delivery room physiological studies, the sample size was small and the intervention was not randomised. In the primary study, the authors compared infants who received surfactant nebulisation and those who did not, finding no difference in lung volumes between groups.^9^

This work by Belting et al. adds to the growing body of literature using EIT to evaluate the respiratory transition at birth.^10,11^ Briefly, EIT is a non-invasive, radiation-free method of measuring real-time, relative changes in regional lung aeration. As the patient interface (a small non-adhesive belt) is placed around the chest wall, it is ideal for assessing spontaneous breathing and non-invasive support, and lacks the limitations of measurement at the airway opening.^12^ In this study, the authors used a common and well-studied EIT measure, change in end-expiratory lung impedance (ΔEELI) to estimate changes in functional residual capacity (FRC) over time.^12^ Unless the EIT impedance signal can be calibrated to a reliable known volume, ΔEELI is reported in arbitrary units (AU/kg) relative to a common reference point, in this case the EELI value just before the mask was placed. Thus, absolute ΔEELI values should not be compared between subjects, but rather the pattern (and not magnitude) of change over time. As the authors recorded continuously within subjects, the time-course change can be considered the relative ΔFRC, consistent with other studies in critical care.^11–13^

The increase in EELI following placement of the NPT in infants unresponsive to mask PPV is an important finding. There is a close association between lung aeration and clinical status at birth.^10,11^ It is reasonable to assume in this group of infants, in whom the clinician decided mask-support was not effective, that initial aeration was poor. The increase in oxygenation and HR after commencing NPT ventilation suggests that the use of an NPT in this selected group of infants aided FRC and may have a role in preventing intubation in preterm infants. It is possible that the gains in FRC seen were not related to the use of a NPT, but rather a product of increased resuscitation, especially pressure levels, or time-based lung aeration. The peak inspiratory pressure used following insertion of the NPT was higher than prior to insertion, confounding the effect of the NPT as an airway adjunct versus the airway pressure itself. In the term neonate, FRC improves with time as lung regions sequentially aerate and become stable enough to support tidal ventilation, an event that occurs once foetal fluid influx from the interstitium during expiration is minimal.^11^ A similar process was recently reported by the authors in preterm infants requiring less resuscitation.^10^ This further highlights the need for more delivery room studies using EIT to better understand these complex physiological and clinical interactions.

Interestingly, the authors noted that the biggest loss in EELI was associated with the period between removing mask support and commencing NPT support. Similar findings have recently been reported between extubation and starting CPAP in preterm infants, leading to increased ventilation heterogeneity.^14,15^ This makes sense as the preterm lung is poorly compliant, highlighting the dependency on positive end-expiratory pressure to maintain aeration. There is a need for interventional studies to inform how best to apply positive end-expiratory pressure in the delivery room and limit the impact of pressure delivery interruptions.

One of the advantages of EIT is the ability to describe lung volume changes regionally as well as globally.^12^ Unfortunately, the authors did not report regional ΔEELI changes or tidal ventilation patterns. Global increases in lung volume will improve oxygenation but if the EELI increases are not uniform, then areas of overdistension and atelectasis may be simultaneously present.^13^ The importance of regional EELI and ventilation patterns in preterm lung injury has been established in preclinical studies, with ventilation and aeration heterogeneity associated with increased injury.^13^ Additionally, small interventional studies in adults with acute respiratory distress syndrome suggest that reducing heterogeneity may improve outcomes. Quantification of ventilation heterogeneity using EIT is simpler than EELI, less impacted by artefact, easier to visualise in real-time, and able to be compared between subjects.^12^

As with any retrospective analysis, caution should be applied to the extrapolation of these study results to clinical practice without more rigorous, prospective investigation. However, this study should serve as promising base for future clinical trials investigating NPT use in preterm resuscitation. The NPT may be a simple, low-cost intervention to avoid early preterm endotracheal intubation and, in turn, reduce the incidence of bronchopulmonary dysplasia. Like face mask and LMA support, the answer may not be in which interface to use but rather how to use it most effectively.
